# High Prevalence of Insufficient Vitamin D Intake and Serum 25-Hydroxyvitamin D in Chinese School-Age Children: A Cross-Sectional Study

**DOI:** 10.3390/nu10070822

**Published:** 2018-06-26

**Authors:** Yan Liu, Xinyi Li, Ai Zhao, Wei Zheng, Mofan Guo, Yong Xue, Peiyu Wang, Yumei Zhang

**Affiliations:** 1 Department of Nutrition and Food Hygiene, School of Public Health, Peking University, Beijing 100191, China; Shyneeliu@163.com (Y.L.); xueyong1986@im.ac.cn (Y.X.); 2 Department of Preventive Medicine, School of Public Health, Peking University, Beijing 100191, China; yaoyao-0703@163.com (X.L.); guomofan@sina.cn (M.G.); 3 Department of Social Medicine and Health Education, School of Public Health, Peking University, Beijing 100191, China; xiaochaai@163.com (A.Z.); yutian84@126.com (W.Z.); wpeiyu138@163.com (P.W.)

**Keywords:** vitamin D intake, 25-hydroxyvitamin D, children, growth and development

## Abstract

Despite the importance of vitamin D in early stages of life, data are lacking on the levels of vitamin D intake and serum 25-hydroxyvitamin D (25-(OH)D) among Chinese school-age children. The aims of this study were to investigate the vitamin D intake and serum 25-(OH)D concentration of children aged 7 to 12 years in China, and to measure the associations between vitamin D status and children’s growth and development parameters. We obtained data on vitamin D intake, serum 25-(OH)D concentration, and anthropometric measurements from a cross-sectional study on school-aged children in China. Multiple linear regression and multivariable logistic regression analyses assessed the associations mentioned above. A total of 563 children (9.5 ± 1.6 years) from six areas of China were included. Among them, 86.1% had a vitamin D intake below the recommended nutrient intake (10 μg/day), and 54.7% had vitamin D deficiency (serum 25-(OH)D <15 ng/mL). We also found that high vitamin D intake was inversely associated with the risk of vitamin D deficiency (OR = 0.836, 95% confidence interval: 0.713, 0.980), and serum 25-(OH)D concentration was positively associated with the intelligence score and body mass index-for-age *Z*-score (BAZ) (*p* < 0.05). Insufficient vitamin D intake and serum 25-(OH)D are prevalent among Chinese school-aged children. A negative association was observed between vitamin D intake and the risk of vitamin D deficiency. Moreover, vitamin D status was positively associated with intelligence and BAZ, which await confirmation in future studies.

## 1. Introduction

Sufficient provision of nutrients in early stages of life is critical to ensure optimal growth, development, and health [1]. However, vitamin D deficiency has emerged as a global public health issue, with particular concern for growing children due to their higher sensitivity to the deficiency than adults [2,3]. Evidence has suggested that, in addition to the fully-investigated influence of vitamin D on the regulation of calcium-phosphorus homeostasis in bone mineralization, vitamin D may be associated with a number of extraskeletal disorders, including food allergies, wheezing, asthma, type 1 diabetes, body fat and autism in children, and rickets, to mention just a few [4].

Besides sun exposure, dietary intake and supplements are also vital sources to maintain or improve vitamin D status, especially in winter [5]. Some reports have suggested that insufficient vitamin D intake was prevalent among young children, even in developed countries [6,7]. However, data on dietary vitamin D intake are still scarce in China, coupled with limited large-scale or national representative data on serum vitamin D status, preventing targeted health education and policymaking for treating vitamin D deficiency.

A clear relationship between dietary vitamin D intake and serum 25-hydroxyvitamin D (25-(OH)D) concentration may help improve the efficiency of nutrition guidance for children. Although some research conducted in other countries has proved a positive correlation between these two factors [8], no relevant evidence exists for Chinese children. Furthermore, despite the importance of vitamin D status for young children being well recognized [4,9,10,11], a definitive conclusion is still lacking on the associations between vitamin D status and children’s growth and development parameters, including intelligence.

We performed this study to: (1) investigate the vitamin D intake and serum25-(OH)D concentration of school-age children from eight areas in China, (2) confirm the association between vitamin D intake and serum 25-(OH)D concentration, and (3) explore the relationship between serum 25-(OH)D concentration and children’s growth and development parameters.

## 2. Materials and Methods

### 2.1. Subjects

Data for this study were collected between November 2011 and November 2012 from healthy children aged 7–12 years old in China using a multi-stage stratified cluster sampling. In the first stage, we selected five cities (Beijing, Suzhou, Guangzhou, Zhengzhou, and Lanzhou), one village in the plains, and one village in the mountainous area (both located in Xingtai, Hebei province) representing various geographic locations including eastern, western, southern, northern, and central China, and different economic development levels including villages and first-tier, second-tier, and third-tier cities via purposive sampling. In the second stage, we randomly chose one large school in each city or village in view of sample size and representativeness. In the last stage, to ensure the coverage of 7–9 years and 10–12 years age groups, we randomly selected one class of grade 1–3 and another class of grade 4–6 children in each school, and surveyed all children in the selected classes. Eligible participants were healthy children aged 7–12 years with normal intelligence and complete information on serum 25-(OH)D concentration. We excluded children with severe diseases (including congenital heart disease, hydrocephalus, infantile paralysis, and thalassemia), deformity, and mental retardation. All participants provided written informed consent forms signed by their legal guardians. This study was approved by the Ethical Committee of the Health Science Center at Peking University (No. IRB00001052-11042).

### 2.2. Assessment of Vitamin D Intake

The school-age children’s vitamin D intake was determined based on the dietary weekday data collected using a 24 h recall. Trained and experienced interviewers coached the parent to carefully recall the time of consumption, types, ingredients, cooking methods, and amounts of all food items, including extra meals. Some food models and a series of pictures of standard bowls and spoons were presented to the parents to help them quantify the food intake of their children. Furthermore, if the children were taking dietary supplements, parents were asked to provide the brand name, manufacturer, and daily dosage of the supplements. Then, we calculated vitamin D intake using a Vitamin D Content of Food Reference Database that we established based on the food and supplements labels and Standard Tables of Food Composition in Japan (2010) [12]. The vitamin D intake status was compared with the dietary reference intakes (DRIs) in the Dietary Guidelines for Chinese Residents (2013) [13]. Vitamin D intake deficiency refers to intake lower than the estimated average requirement (EAR, 8 μg/day for all children), insufficiency refers to intake higher than EAR yet lower than the recommended nutrient intake (RNI, 10 μg/day for all children), and intake higher than RNI is considered to be sufficient. In addition, we considered vitamin D intake beyond the tolerable upper intake level (UL, 45 μg/day for children aged 7–10 years, 50 μg/day for children aged 11–12 years) as excessive vitamin D consumption.

### 2.3. Assessment of Blood Indicators

Fasting venous blood samples of children were collected in the morning and transported in ice boxes with ice packs to Beijing Lawke Health Laboratory (Beijing, China) for assessment of serum 25-(OH)D concentration and calcium concentration.

Serum 25-(OH)D concentration (the total of 25-(OH)D2 and 25-(OH)D3) was measured with the liquid chromatography-tandem mass spectrometry (LC-MS/MS) method, the standard for 25-(OH)D determination, by using a high performance liquid chromatograph (Agilent 1100; Agilent Technologies Inc., Santa Clara, CA, USA) and a mass spectrometer (API4000Q trap; AB SCIEX LLC., Redwood City, CA, USA). The lower limits of detection were 2 and 1 ng/mL for 25-(OH)D2 and 25-(OH)D3, respectively. We adopted the criteria supported by the American Academy of Pediatrics to categorize the vitamin D status of children (serum 25-(OH)D <5 ng/mL, severe deficiency; <15 ng/mL, deficiency; 15–20 ng/mL, insufficiency; 20–100 ng/mL, sufficiency; 32–100 ng/mL, optimal level; >100 ng/mL, overdose; >150 ng/mL, intoxication) [14]. The concentration of calcium in blood was measured with an AA-7010 atomic absorption spectrophotometer (Bohui Innovation Co. Ltd., Beijing, China).

### 2.4. Assessment of Growth and Development

Trained researchers conducted the children’s anthropometry measurements in a comfortable examination area with the children wearing minimal clothing. Height was measured within 0.1 cm, and weight was measured accurate to 0.1 kg. Both measurements were recorded twice and the mean values were calculated. body mass index (BMI) was defined as weight/height^2^ (kg/m^2^). With reference to the World Health Organization (WHO) Growth Standards, we calculated the height for age *Z*-score (HAZ), weight for age *Z*-score (WAZ), and BMI-for-age *Z*-score (BAZ) using WHO AnthroPlus. We also measured the speed of sound (SOS) of the distal 1/3 radius using Omnisense TM 7000P ultrasound bone sonometer (Sunlight, Rehovot, Israel).

Intelligence was assessed by trained interviewers using the Chinese version of the Wechsler Intelligence Scale for Children (C-WISC), which has shown good reliability and validity for measuring general intelligence of school-age children in China [15]. The C-WISC contains verbal and performance subtests that yield the verbal intelligence quotient (VIQ) and performance intelligence quotient (PIQ) scores, which together produce a full-scale intelligence quotient (FIQ) score.

#### Assessment of Covariates

Investigators administered a structured interview of school-age children’s parents to collect sociodemographic information, such as child’s date of birth and sex, as well as height, weight, educational level of parents, and household income per capita. The season of the observations and children’s frequency and duration of physical activity both at school and at home 24 h before the survey were also collected.

### 2.5. Statistical Analysis

We applied mean ± SD to describe normally distributed continuous variables, and performed Student’s *t*-test or one-way analysis of variance (ANOVA) with the least squares differences (LSD) method to test differences among groups. We present non-normally distributed continuous data with the median (25th percentile, 75th percentile) and conducted a Mann-Whitney U test or Kruskal-Wallis H test with a paired comparison method for intergroup comparison. Categorical data were described using frequency, constituent ratio, or percentage, and examined differences between groups using Pearson’s χ^2^ test with the Bonferroni method.

We divided participants into quartile groups according to their vitamin D intake (Q1–Q4). The multivariable logistic regression model was used to analyze the association between vitamin D intake (Q1–Q4, rank variable) and vitamin D deficiency (severe deficiency included), with Models 0–3 controlling covariates at different levels (Model 0: no adjustment). The multiple linear regression model was applied to investigate the associations between serum 25-(OH)D concentration (continuous variable) and growth and development indexes with different covariate adjustments in Models 1 and 2. All analyses were performed using statistical software package IBM SPSS Statistics Version 19.0. Statistical significance was determined at *p* < 0.05 (two-tailed tests).

## 3. Results

### 3.1. Participant Characteristics

After excluding six children with missing data in basic or dietary information, a total of 563 children aged 7–12 years participated in the analysis. Basic characteristics of the children by sex are provided in Table 1. Boys showed higher BAZ than girls. No significant sexual difference was observed between the groups for other basic characteristics (Table 1).

### 3.2. Vitamin D Intake

The median (25th percentile, 75th percentile) vitamin D intake of all participants was 1.1 (0.5, 2.2) μg/day. Of the children’s vitamin D intake, 84.2%, 86.1%, and 8.9% were below EAR, below RNI, and beyond UL, respectively. Parents’ education level and family income were important factors associated with children’s vitamin D intake status. The higher the parents’ education level and the household income per capita, the higher the vitamin D intake and the lower the percentage of vitamin D intake deficiency and insufficiency. No significant differences were found between sexes and different age groups in terms of these medians and percentages (Table 2).

### 3.3. Vitamin D Status

The average serum 25-(OH)D concentration of the participant children was 15.0 ± 7.9 ng/mL. A total of 3.2%, 54.7%, and 76.9% of the children’s serum 25-(OH)D concentrations were below 5 ng/mL, 15 ng/mL, and 20 ng/mL, respectively. Only 23.1% of children reached a sufficient level of serum vitamin D. Among all the children studied, sex, age, household income per capita, vitamin D intake level, season of survey, and latitude of survey location were all relevant to children’s serum vitamin D status. However, significant differences between both the different education levels for the mother and father were not observed in our study regarding the serum vitamin D status (Table 3).

### 3.4. Association between Vitamin D Intake and Vitamin D Status

After adjustment of possible influential factors at different levels in multivariable logistic regression for Models 0–3 (Model 0: no adjustment), we found that high levels of vitamin D intake consistently appeared to be the protective factor of vitamin D deficiency, as a higher quartile of vitamin D intake was associated with a 16.4% reduction in risk of vitamin D deficiency (Model 3; Table 4).

### 3.5. Associations between Serum Vitamin D Concentration and Growth and Development of Children

To analyze the relationships between serum 25-(OH)D concentration and distal radius SOS and intelligence score, we adjusted covariates differently in multilevel linear regression Model 1 and Model 2 and found serum 25-(OH)D concentration was positively associated with the intelligence score (Table 5). In terms of *Z*-scores of height, weight, and BMI of children, we adjusted covariates differently in multilevel linear regression Model 1 and Model 2 (Model 1: no adjustment). Serum 25-(OH)D concentration was found to be positively associated with WAZ and BAZ in Model 1, whereas the former relationship disappeared in Model 2 (Table 5).

## 4. Discussion

In this study, we found that more than four-fifths of children had low vitamin D intake (84.2% below EAR, 86.1% below RNI) indicating that inadequate vitamin D intake is an extremely severe problem among school-age children. However, we also found that 8.9% children had excessive vitamin D intake. According to the database of the 24 h dietary recall, tremella and agaric were the main reasons for excessive vitamin D intake. In addition, dietary supplements, fish, eggs, milk and milk products fortified with vitamin D, livestock and poultry meat, mushroom, and some other food added with vitamin D also contributed to this result. Thus, parents should pay more attention to children’s diet to prevent both inadequate and excessive vitamin D intake.

Worldwide, researchers have reported vitamin D deficiency as a major public health issue, even in regions with abundant sun exposure. However, a striking lack of data exists for the vitamin D status of children. Based on available data, vitamin D insufficiency was observed in 16, 35, 31, and 58% children in the United States, United Kingdom, New Zealand, and Belgium, respectively, whereas the prevalence reached 72% and up to 95% in Malaysia and Afghanistan, respectively [3]. This study revealed that the average serum 25-(OH)D level of Chinese school-age children was 15.0 ± 7.9 ng/mL, which is far below the desirable value and approximately 8 out of 10 (76.9%) surveyed children were diagnosed with vitamin D insufficiency according to criteria endorsed by the American Academy of Pediatrics [14].

Sun exposure is an ideal source of vitamin D, however, other factors may decrease exposure, including sun-avoiding behavior, skin pigmentation, air pollution, high latitude, and other factors that influence vitamin D production [4,16]. Thus, people seem to depend more on dietary and supplemental sources to achieve optimal serum 25-(OH)D concentration. In line with previous studies in many other countries [8], we found that high vitamin D intake is a protective factor of vitamin D deficiency among school-age children. This result suggests that children should be appropriately encouraged to increase vitamin D intake.

Vitamin D serves many physiological functions given the wide distribution of vitamin D receptors [9,10,17,18]. One of the major physiologic functions of vitamin D is to regulate bone metabolism by adjusting serum calcium and phosphorus concentrations [4]. Whereas most previous studies found adequate vitamin D to be beneficial for bone health [19,20,21,22], associations between serum vitamin D concentration and parameters closely related to bone growth such as height, SOS, and HAZ were not observed in this study. Evidence from a comparative meta-analysis suggested that sufficient calcium intake lay the basis for vitamin D to exert its positive effects on bone [23]. Therefore, we assumed the low calcium intake of the participants (351.5 ± 233.8 mg/d) might hinder the effect of vitamin D on bone and related parameters. Additionally, we suggested co-supplementation of vitamin D and calcium to optimize clinical efficacy.

Another important role of vitamin D is in the brain [24,25]. Our findings demonstrated the positive association between serum 25(OH)D concentration and intelligence score. Evidence from animal tests provided the biological plausibility of this finding. For instance, Eyles et al. demonstrated that rats born to vitamin D_3_ deficient mothers had profound alterations in the brain [24]. Furthermore, Brown et al. found that the addition of vitamin D_3_ significantly decreased the percentage of cultured hippocampal cells undergoing mitosis and increased the production of neurite outgrowth and nerve growth factor (NGF) [26]. Similarly, vitamin D might enhance cognitive functioning in older adults [27,28,29,30,31].

A negative correlation between obesity and serum vitamin D concentration was first observed in rats in 1971, which was thought to be caused by a large amount of vitamin D stored in adipose tissue in obese individuals [32]. Since then, a growing body of epidemiological evidence has affirmed this finding in children [33,34]. Therefore, we investigated the association between serum 25-(OH)D concentration and WAZ and BAZ. Interestingly, we found a positive correlation between serum 25-(OH)D concentration and BAZ after adjusting for covariates, although some studies reported similar results [35,36]. An explanation for this finding is that BAZ is not a good representative of obesity in school-age children. The use of BAZ fails to differentiate fat from lean body mass, the growth of which consists of the majority of weight gain for healthy children in a period of rapid growth and development. Thus, we hypothesized vitamin D might be crucial for the increase in lean body mass in children and further studies in this area were warranted.

To the best of our knowledge, this is the first study that reported the dietary vitamin D intake among children aged 7–12 years in China. This is also the first time the relationship between dietary vitamin D intake and serum 25-(OH)D levels in this population have been analyzed. Moreover, since serum 25-(OH)D levels were detected by LC-MS/MS, which is the standard, we confirmed the measurement accuracy of vitamin D status among Chinese children.

Limitations of this study should also be considered. First, since data on vitamin D content in China Food Composition are still lacking, we referred to the Standard Tables of Food Composition in Japan (2010) [12] to calculate the vitamin D intake of Chinese children, which cannot be fully applied to China due to the heterogeneity in food types between the two countries despite the similarities they share. Second, providing a precise estimate of an individual’s usual vitamin D intake is difficult for a one-day 24 h dietary recall only from parents, although a face-to-face interview by trained researchers with the aid of food models was conducted to reduce this error. However, as was performed in the present study, the data can be useful to assess group medians. Third, vitamin D status between seasons might be confounded by the different latitudes of the survey locations. Most blood samples (67.1%) collected in summer belonged to children living in high latitudes, whereas most blood samples (72.8%) collected in winter belonged to children living in low latitudes area. Fourth, since the criteria for categorizing the serum 25-(OH)D concentration corresponding to the Dietary Reference Intakes (DRIs) from the Dietary Guidelines for Chinese Residents (2013) are still lacking in China, we chose the cut-off points supported by American Academy of Pediatrics, which may have affected the assessment of vitamin D status of the subjects. Fifth, outdoor activity time may also have affected the results, which was not available in our study.

## 5. Conclusions

In summary, this observational study revealed a high prevalence of insufficient vitamin D intake and serum 25-(OH)D level among school-age children in China, and dietary vitamin D intake was highly associated with vitamin D status in this population. Serum 25-(OH)D concentration was also found to be positively associated with intelligence and BAZ, which awaits confirmation by future studies. These findings suggest that strategies regarding the prevention and treatment of vitamin D deficiency in children are urgently needed.

## Figures and Tables

**Table 1:** Basic characteristics of participants by sex.

	Total	Boys (*n* = 291)	Girls (*n* = 272)	*p* Value
Characteristics of child (mean ± SD ^1^)				
Age (year)	9.5 ± 1.6	9.5 ± 1.6	9.6 ± 1.6	0.359
Distal radius SOS ^1^ (m/s)	3697.2 ± 126.7	3696.5 ± 131.2	3698.0 ± 122.0	0.887
Intelligence score	100.7 ± 15.4	101.6 ± 16.5	99.8 ± 14.2	0.155
HAZ ^1^	0.3 ± 1.1	0.4 ± 1.1	0.2 ± 1.1	0.090
WAZ ^1^ (7–9 years)	0.3 ± 1.3	0.5 ± 1.3	0.2 ± 1.2	0.058
BAZ ^1^	0.0 ± 1.5	0.2 ± 1.5	−0.1 ± 1.4	0.007
Physical activity (min/day)	94.0 ± 81.3	95.7 ± 86.6	92.3 ± 75.2	0.629
Blood calcium (mmol/L)	1.7 ± 0.1	1.7 ± 0.1	1.7 ± 0.1	0.651
Characteristics of mother (mean ± SD ^1^)				
Height (cm)	161.3 ± 4.7	161.5 ± 4.9	160.1 ± 4.6	0.366
Weight (kg)	58.2 ± 8.0	58.0 ± 8.1	58.4 ± 7.8	0.580
BMI ^1^ (kg/m^2^)	22.4 ± 2.9	22.3 ± 3.0	22.5 ± 2.8	0.366
Education level (*n* (%))				
Of mother				0.399
Middle school or below	321 (57.0)	169 (58.1)	152 (55.9)	
High school	183 (32.5)	91 (31.3)	92 (33.8)	
College or above	53 (9.4)	26 (8.9)	27 (9.9)	
Unclear	6 (1.1)	5 (1.7)	1 (0.4)	
Of father				0.568
Middle school or below	252 (44.8)	137 (47.1)	115 (42.3)	
High school	232 (41.2)	118 (40.5)	114 (41.9)	
College or above	73 (13.0)	33 (11.3)	40 (14.7)	
Unclear	6 (1.1)	3 (1.0)	3 (21.1)	
Household income per capita (Yuan) (*n* (%))				0.187
<2000	257 (45.6)	122 (41.9)	135 (49.6)	
2000–4000	133 (23.6)	70 (24.1)	63 (23.2)	
>4000	82 (14.6)	44 (15.1)	38 (14.0)	
Unclear	91 (16.2)	55 (18.9)	36 (13.2)	
Season of survey (*n* (%))				0.461
Summer (May, July)	258 (45.8)	129 (44.3)	129 (47.4)	
Winter (March, November, December)	305 (54.2)	162 (55.7)	143 (52.6)	
Latitude of survey spot (*n* (%))				0.823
≤37°	307 (54.5)	160 (55.0)	147 (54.0)	
>37°	256 (45.5)	131 (45.0)	125 (46.0)	

*p* values were calculated by Student’s *t*-tests for continuous variables and Pearson’s χ^2^ test for categorical variables.^1^ SD, standard deviation; SOS, speed of sound; HAZ, WAZ, and BAZ refer to height for age *Z*-score, weight for age *Z*-score, and BMI-for-age *Z*-score of children, respectively; BMI, Body Mass Index.

**Table 2:** Vitamin D intake status of school-age children by relevant variables.

		Vitamin D Intake (μg/Day)		<EAR^3^		<RNI^3^		>UL^3^	
		Median (25th, 75th)	*p* Value	Percentage (%)	*p* Value	Percentage (%)	*p* Value	Percentage (%)	*p* Value
Sex			0.980		0.488		0.368		0.126
	Male	1.1 (0.5, 2.4)		242 (83.2)		247 (84.9)		31 (10.7)	
	Female	1.1 (0.5, 2.2)		232 (85.3)		238 (87.5)		19 (7.0)	
Age (year)			0.818		0.205		0.505		0.742
	7–9	1.1 (0.5, 2.1)		253 (86.1)		256 (87.1)		25 (8.5)	
	10–12	1.1 (0.5, 2.7)		221 (82.2)		229 (85.1)		25 (9.3)	
Education level of mother			<0.001		0.076		0.125		0.122
	Middle school or below	0.9 (0.4, 1.8) ^1^		279 (86.9)		284 (88.5)		22 (6.9)	
	High school	1.4 (0.6, 3.5) ^2^		145 (79.2)		150 (82.0)		22 (12.0)	
	College or above	1.5 (0.9, 3.3) ^2^		44 (83.0)		45 (84.9)		6 (11.3)	
Education level of father			<0.001		0.001		0.010		0.075
	Middle school or below	0.9 (0.2, 1.8) ^1^		228 (90.5) ^1^		229 (90.9) ^1^		15 (6.0)	
	High school	1.3 (0.7, 4.8) ^2^		181 (78.0) ^2^		189 (81.5) ^2^		27 (11.6)	
	College or above	1.8 (0.8, 3.9) ^2^		59 (80.8) ^2^		61 (83.6) ^1,2^		8 (11.0)	
Household income per capita (Yuan)			<0.001		0.001		0.005		0.203
	<2000	0.9 (0.4, 1.8) ^1^		231 (89.9) ^1^		232 (90.3) ^1^		19 (7.4)	
	2000–4000	1.3 (0.5, 6.2) ^2^		102 (76.7) ^2^		108 (81.2) ^2^		17 (12.8)	
	>4000	1.7 (0.8, 4.6) ^2^		64 (78.0) ^2^		64 (78.0) ^2^		9 (11.0)	

*p* values were calculated by Mann-Whitney U test or Kruskal-Wallis H test for continuous variables and Pearson’s χ^2^ test for categorical variables. ^1,2^ Different letters in same column indicates significant differences between groups, *p* < 0.05. ^3^ EAR, average requirement; RNI, recommended nutrient intake; UL, tolerable upper intake level.

**Table 3:** Serum vitamin D status of school-age children by relevant variables.

		Concentration (ng/mL)		<5 (ng/mL)		<15 (ng/mL)		<20 (ng/mL)	
		Mean ± SD ^3^	*p* Value	Percentage (%)	*p* Value	Percentage (%)	*p* Value	Percentage (%)	*p* Value
Sex			0.041		0.739		0.223		0.018
	Male	15.7 ± 8.6		10 (3.4)		152 (52.2)		212 (72.9)	
	Female	14.3 ± 7.0		8 (2.9)		156 (57.4)		221 (81.3)	
Age (year)			0.025		0.773		0.012		0.068
	7–9	15.7 ± 8.2		10 (3.4)		146 (49.7)		217 (73.8)	
	10–12	14.2 ± 7.4		8 (3.0)		162 (60.2)		216 (80.3)	
Education level of mother			0.053		0.329		0.125		0.072
	Middle school or below	14.5 ± 8.3		13 (4.0)		188 (58.6)		257 (80.1)	
	High school	16.1 ± 7.3		3 (1.6)		90 (49.2)		131 (71.6)	
	College or above	13.9 ± 6		2 (3.8)		29 (54.7)		43 (81.1)	
Education level of father			0.160		0.669		0.109		0.117
	Middle school or below	14.5 ± 8.7		10 (4.0)		150 (59.5)		202 (80.2)	
	High school	15.7 ± 7.3		6 (2.6)		116 (50)		169 (72.8)	
	College or above	14.2 ± 6		2 (2.7)		40 (54.8)		59 (80.8)	
Household income per capita (Yuan)			0.002		0.395		0.011		0.012
	<2000	13.4 ± 7.2 ^1^		12 (4.7)		163 (63.4) ^1^		215 (83.7) ^1^	
	2000–4000	16.2 ± 8.3 ^2^		3 (2.3)		65 (48.9) ^2^		95 (71.4) ^2^	
	>4000	15.1 ± 6.7 ^1,2^		2 (2.4)		42 (51.2) ^2^		61 (74.4) ^1,2^	
Vitamin D intake			0.157		0.726		0.034		0.387
	<RNI	14.8 ± 7.8		15 (3.1)		274 (56.5)		376 (77.5)	
	>RNI	16.2 ± 8.2		3 (3.8)		34 (43.6)		57 (73.1)	
Season of survey			<0.001		0.001		<0.001		<0.001
	Summer (May, July)	12.9 ± 6.2		15 (5.8)		163 (63.2)		223 (86.4)	
	Winter (March, November, December)	16.8 ± 8.7		3 (1.0)		145 (47.5)		210 (68.9)	
Latitude of survey location			<0.001		0.001		<0.001		<0.001
	≤37°	16.6 ± 7.9		3 (1.0)		138 (45.0)		218 (71.0)	
	>37°	13.2 ± 7.5		15 (5.9)		170 (66.4)		215 (84.0)	

*p* values were calculated by Student’s *t*-test or one-way ANOVA for continuous variables and Pearson’s χ^2^ test a for categorical variables. ^1,2^ Different letters in same column indicates significant differences between groups, *p* < 0.05. ^3^ SD, standard deviation.

**Table 4:** Association between vitamin D intake and vitamin D status.

	*β*	SEM ^1^	*Wals*	*p* Value	OR ^1^	95% CI ^1^
Model 0	−0.272	0.075	13.100	<0.001	0.762	0.658–0.883
Model 1	−0.265	0.076	12.230	<0.001	0.767	0.661–0.890
Model 2	−0.233	0.078	8.856	0.003	0.792	0.680–0.924
Model 3	−0.179	0.081	4.868	0.027	0.836	0.713–0.980

Associations were examined using multivariable logistic regression. ^1^ SEM, standard error of mean; OR, odds ratio; CI, confidence interval. Model 0 was the crude analysis; model 1 was adjusted for sex and age; model 2 was adjusted for sex, age, education level of parents and, household income per capita; and model 3 adjusted for sex, age, education level of parents, household income per capita, season of survey, and latitude of the location.

**Table 5:** Associations between vitamin D concentration and growth and development parameters.

	**Model 1 ^2^**						**Model 2 ^3^**					
	**R Square Change**	**Adjusted R Square**	***β***	**SEM**	***t* Value**	***p* Value**	**R Square Change**	**Adjusted R Square**	***β***	**SEM**	***t* Value**	***p* Value**
Distal radius SOS ^1^ (m/s)	0.002	0.003	−0.325	0.688	−0.471	0.638	0.006	−0.005	−0.096	0.719	−0.133	0.894
Intelligence score	0.019	0.014	0.207	0.083	2.482	0.013	0.166	0.157	0.161	0.078	2.060	0.040
	**Model 1 ^4^**						**Model 2 ^5^**					
	**R Square Change**	**Adjusted R Square**	***β***	**SEM**	***t* Value**	***p* Value**	**R Square Change**	**Adjusted R Square**	***β***	**SEM ^1^**	***t* Value**	***p* Value**
HAZ ^1^	0.002	0.000	0.006	0.006	1.081	0.280	0.070	0.061	0.008	0.006	1.273	0.204
WAZ ^1^ (7–9 years)	0.029	0.025	0.026	0.009	2.903	0.004	0.101	0.084	0.014	0.010	1.403	0.162
BAZ ^1^	0.021	0.019	0.027	0.008	3.429	0.001	0.072	0.063	0.024	0.008	2.952	0.003

Associations were examined by using multilevel linear regression. ^1^ SOS, speed of sound; HAZ, WAZ and BAZ refer to height for age *Z*-score, weight for age *Z*-score, and BMI-for-age *Z*-score of children, respectively; SEM, standard error of mean. ^2^ Model 1 adjusted for sex and age. ^3^ Model 2 adjusted for sex, age and household income per capita (for distal radius SOS: plus adjustment of blood calcium and overall duration of physical activity; for intelligence score, plus adjustment of education level of parents). ^4^ Model 1 was the crude analysis. ^5^ Model 2 adjusted for household income per capita, blood calcium, and overall duration of physical activity(for HAZ, plus adjustment of height of mother; for WAZ, plus adjustment of weight of mother; and for BAZ, plus adjustment of BMI of mother).
